# Potential Role of Superoxide Dismutase 3 (SOD3) in Resistance to Influenza A Virus Infection

**DOI:** 10.3390/antiox12020354

**Published:** 2023-02-02

**Authors:** Feimin Chen, Liurong Chen, Jinlong Liang, Zhixuan Chen, Chunyu Zhang, Zhengyin Zhang, Jie Yang

**Affiliations:** 1 NMPA Key Laboratory for Research and Evaluation of Drug Metabolism, Guangdong Provincial Key Laboratory of New Drug Screening, School of Pharmaceutical Sciences, Southern Medical University, Guangzhou 510515, China; 2 Department of Pharmacy, Xiangyang Central Hospital, Affiliated Hospital of Hubei University of Arts and Science, Xiangyang 441000, China

**Keywords:** SOD3, influenza A virus, ROS, replication, inflammatory response

## Abstract

Influenza A virus infection induces the production of excessive reactive oxygen species (ROS). Overproduction of ROS can overwhelm the antioxidant defense system, leading to increasing intensive oxidative stress. However, antioxidant defense against oxidative damage induced by influenza A virus infection, and in particular the significance of the SOD3 response in the pathogenesis of influenza virus infection, has not been well characterized. Here, we investigated the potential role of SOD3 in resistance to influenza A virus infection. In this study, SOD3, as an important antioxidant enzyme, was shown to be highly elevated in A549 cells following influenza A virus infection. Furthermore, inhibition of SOD3 impacted viral replication and virulence. We found that SOD3 disrupts IAV replication by impairing the synthesis of vRNA, whereas it did not affect viral ribonucleoprotein nuclear export. In addition, overexpression of SOD3 greatly reduced the levels of ROS caused by influenza A virus infection, regulated the inflammatory response to virus infection by inhibiting the phosphorylation of p65 of the NF-κB signaling pathway, and inhibited virus-induced apoptosis to a certain extent. Taken together, these findings indicate that SOD3 is actively involved in influenza A virus replication. Pharmacological modulation or targeting of SOD3 may pave the way for a novel therapeutic approach to combating influenza A virus infection.

## 1. Introduction

Influenza is a contagious respiratory illness caused by influenza viruses, which have posed substantial challenges to public health worldwide. In particular, influenza A virus (IAV) has caused massive recurring epidemics, with significant morbidity and occasional pandemics. The continuing outbreak of highly pathogenic H5N1 avian influenza, with its high fatality rate, has raised serious concerns [1]. A novel avian-origin H7N9 IAV was initially detected in China in 2013 and continues to circulate in this region, posing a significant threat to human health [2]. In addition, new recombinant avian influenza viruses have developed consistently over the last two years, indicating that we are on the verge of a pandemic. The genetic variants of the influenza virus present serious challenges to combating this ongoing pandemic. Currently, effective treatments are still lacking because of viral drug resistance or inadequately effective vaccines, especially against the highly pathogenic influenza virus. Thus, there is an urgent need to develop new therapeutic strategies, particularly based on host factors that are critical for influenza virus infection.

A large number of host factors have been increasingly recognized that can directly or indirectly affect viral infection, such as various cellular enzymes [3,4]. Pro-antioxidant balance is critical for the host’s normal functioning, whereas oxidative stress caused by viral stimulation disrupts the intracellular redox balance, leading to a significant decrease in antioxidant defenses [5,6,7]. For this reason, the establishment of special environments in the host is very important to resisting virus infection. There is growing evidence that oxidative stress is a characteristic of some viral infections, such as RSV, HCV, influenza virus, and SARS-CoV-2, and one of the most effective strategies for combating virus infection is the use of antioxidants [7,8,9,10]. Endogenic genes with antioxidant effects, including superoxide dismutases (SODs), catalase, and glutathione peroxidase, participate in cellular defenses against oxidative damage to maintain cellular redox homeostasis.

Comprehensive studies have revealed superoxide dismutases (SODs) are an important antioxidant enzyme family that protects organisms from oxidative stress [11]. They are crucial for handling the reactive oxygen species (ROS) generated during stress because unbalanced high levels of ROS in living organisms can cause damage. SODs can catalytically convert superoxide radicals to hydrogen peroxide (H_2_O_2_), thus playing a determinant role in the equilibrium between the production and the scavenging of ROS [12,13]. Alongside adaptability to the overload environment, SODs as the primary endogenous defense can improve the host’s resistance to external disease-inducing viral infection [14,15]. The role of SODs in tumor biology has been extensively studied, and it has been found that they are typically downregulated [16,17]. Recent evidence has suggested SODs may boost antioxidant defenses against virus infections [18]. However, how SODs are directly involved in host defenses against viral infections deserves further investigation.

SODs are classified into three distinct groups in eukaryotes: SOD1 (CuZn-SOD), SOD2 (Mn-SOD), and SOD3 (EC-SOD) [19,20]. Each subunit of SOD3 consists of a copper and a zinc atom, and they are synthesized by a signal peptide that directs this enzyme exclusively to extracellular spaces [19,21]. Previous studies have shown that SOD3 (EC 1.15.1.1) as an extracellular superoxide dismutase has multiple characteristics, including growth regulatory, anti-inflammatory, anti-oxidative, and anti-apoptotic activities [19]. Furthermore, SOD3 is involved in the pathogenesis of multiple human diseases. For example, depletion of SOD3 contributes to inflammation and lung fibrosis in mice [22,23,24]. However, studies devoted to SOD3 functions in response to IAV stimulation are limited.

Since oxidative stress is a key risk factor during IAV infection, in the present study, we investigated the potential role of SOD3 in resistance to IAV infection. SOD3 was shown to be substantially elevated following IAV infection, both in vitro and in vivo. We then investigated the potential mechanism of SOD3 in influenza pathogenesis and found SOD3 was closely related to the process of viral replication.

## 2. Materials and Methods

### 2.1. Cells and Viruses

We grew 293T (human embryonic kidney) cells and MDCK (Madin Darby canine kidney) cells (ATCC, Manassas, VA, USA) in Dulbecco’s modified Eagle’s medium (DMEM) supplemented with 10% FBS and 1% penicillin/streptomycin. A549 cells (ATCC, Manassas, VA, USA) were cultured in RPMI 1640 medium supplement with 10% FBS and 1% penicillin/streptomycin. IAV strains including A/Puerto Rico/8/34 (H1N1) (PR8 strain) and A/Aichi/2/68 (H3N2) used in this study were amplified by 8-day-old chicken embryos and kept at −80 °C.

### 2.2. Plasmids

Plasmids pHW2K-NP, pHW2K-PA, pHW2K-PB1, pHW2KPB2, and pPolI-Fluc (firefly luciferase reporter plasmid) used in Mini-replicon system were kindly presented by Professor Bojian Zheng (University of HongKong, HK, China). Renilla luciferase plasmid (hRluc-TK) was purchased from Promega (Beijing, China). The SOD3-encoding plasmid was purchased from GeneCopoeia (Guangzhou, China). The siRNA against SOD3, as well as the control RNA, were purchased from GenePharma (Shanghai, China).

### 2.3. Antibodies

Primary antibody against SOD3 was obtained from Enzo Life Sciences (Plymouth Meeting, PA, USA). The specific primary antibodies NP and polymerase PB2 were purchased from Genetex (USA). NF-kappaB p65, SOD3, phospho-NF-κB p65, p38 MAPK, phospho -p38 MAPK, Nrf2, and anti-Flag were purchased from Cell Signaling Technology (Danvers, MA, USA). Anti-mouse/rabbit secondary antibodies labeled with horseradish peroxidase were bought from Cell Signaling Technology. In indirect immunofluorescence assay, NP primary antibody was bought from Genetex (San Antonio, TX, USA), anti-rabbit-FITC secondary antibody was bought from Absin (Shanghai, China), and Hochest was offered by Sigma-Aldrich.

### 2.4. Viral Infection

Confluent A549 cells were washed twice with PBS and infected with IAV at the indicated multiplicity of infection (MOI). After 1 h incubation at 37 °C, cells were washed twice with PBS and cultured in fresh media, and 1 mg/mL of tolylsulfonyl phenylalanyl chloromethyl ketone (TPCK) trypsin (Worthington Biochemicals, Tryp-MEM) at 37 °C. At the times indicated, viral titer in the supernatants was determined by plaque assay, total proteins were collected for Western blot analysis, and total cellular RNA was extracted from cells for analysis of related gene expression. After 6 h post infection, cell monolayers were fixed and analyzed by immunofluorescent staining with an antibody specific to influenza A nucleoprotein (DAKO Imagen).

### 2.5. ROS Measurement

Intracellular ROS production was detected using 2′-7′-dichlorodihydrofluorescein diacetate (DCFH-DA) (Sigma-Aldrich), described previously with some modifications [25]. Briefly, A549 cells were cultured in the six-well plates and infected with IAV (PR8, MOI = 1). The infected A549 cells were trypsinized at an indicated timepoint after IAV infection followed by incubation with 5 μM DCFH-DA-containing serum-free medium for 20 min at 37 °C. The cells were analyzed by BD FACS Canto II flow cytometer (San Jose, CA, USA). The data were analyzed using FlowJo software (Treestar, San Carlos, CA, USA). At least 10,000 cells were analyzed for each sample. Mean fluorescence intensity is reported as percent increase over control cells.

### 2.6. Plaque Assay

Plaque assay was performed as previously described [26]. Briefly, MDCK cell monolayers were inoculated with the culture supernatant of virus-infected cells for 1 h at 37 °C. Virus inoculums were removed, and infected cells were overlaid with 2 × DMEM medium into equivalent microcrystalline cellulose solution containing additional 1 μg/mL TPCK-trypsin. Cells were incubated for 72 h and then plaques were visualized by staining with 2% crystal violet.

### 2.7. Determination of Malondialdehyde (MDA) and Superoxide Dismutase (SOD) Detection

The production of malondialdehyde (MDA) and the activities of total superoxide dismutase (T-SOD) were determined using commercially available kits (Jiancheng Bioengineering Institute, Nanjing, China). Briefly, A549 cells were infected with IAV (PR8). After incubation for 48 h, the cell lysates were gathered and centrifuged at 1000× *g* for 10 min at −4 °C. The supernatants of cell lysates were used in the antioxidant assays in accordance with the manufacturer’s protocol.

### 2.8. The Activity of NOX Oxidase

The activity of NOX oxidase was determined using commercially available kit (Solarbio, Beijing, China) by a microplate reader (GENios Pro, TECAN, Triangle Park, NC, USA). Briefly, A549 cells were infected with the different MOI of IAV (PR8, MOI = 0.01, 0.1, 1, 10). After incubation for 24 h, cells were lysed, and NOX enzyme activity was determined according to manufacturer’s instructions. A549 cells not infected with IAV were used as the blank control group. H_2_O_2_-treated cells were used as the positive control group. The enzyme activity was expressed as units per milligram protein (U/mg prot).

### 2.9. siRNA Transfection

A549 cells were transiently transfected with siRNA-targeted SOD3 (GenePharma, China) or nonspecific siRNA at the final concentration of 50 nM by using Lipofectamine 3000 (Invitrogen, Carlsbad, CA, USA) according to the manufacturer’s instructions. After incubation for 4 h, the medium was replaced by fresh medium containing 10% FBS and cells were infected with PR8 virus at an MOI of 1. After 24 h transfection, total proteins and messenger RNA (mRNA) were extracted for subsequent experiments. The sequences of siRNA were shown as follows: Forward: 5′CAGCAGAACUGUACCUGUUUU3′; Reverse: 3′UUGUCGUCUUGACAUGGACAA5′.

### 2.10. SOD3 Overexpression

A549 cells were transfected with 1 μg of pHA-SOD3, or vector plasmid for the control group, by using Lipofectamine 3000 (Invitrogen, Waltham, MA, USA) according to the reagent protocol. Subsequently, the medium was replaced by fresh medium containing 10% FBS. After 24 h transfection, cells were infected with PR8 virus at an MOI of 1, as described above. The collected supernatant at different timepoints was determined by plaque assay, and total proteins and mRNA were extracted for subsequent experiments.

### 2.11. Quantitative Real-Time PCR (qRT-PCR)

Expression levels of the genes were analyzed by using qRT-PCR. Total RNA of the virus-infected cells was extracted by TRIzol reagent and used for qRT-PCR, as previously described [27]. Afterward, 1 µg of RNA was reversely transcribed. Gene expression was monitored through SyBr green-based RT-PCR (Invitrogen) in the reaction mixture by using an ABI7500 system (Applied Biosystems, Foster, CA, USA). Gene expression was quantified via 2^−ΔΔCT^ method using 7500 software and GADPH as reference gene. The mRNA levels were expressed as relative gene expression compared with control. The primers used in this study are listed in Table S1.

Additionally, the expression of viral RNA (vRNA), cRNA, and mRNA was determined by qRT-PCR, as previously described [28]. The total RNA was incubated with DNase I at 37 °C for 30 min to remove possible plasmid DNA contamination, and DNase I was inactivated by incubation at 85 °C for 15 min. Reverse transcription (RT) was conducted using strand- and sense-specific oligonucleotides for vRNA (5′-AGCGAAAGCAGG-3′ and 5′-AGCAAAAGCAGG-3′), cRNA (5′-AGTAGAAACAAGG-3′), and mRNA [oligo (DT)]. The RT reaction mixture also included a glyceraldehyde-3-phosphate dehydrogenase (GAPDH)-specific primer (5′-GAAGATGGTGATGGGATTTC-3′) for vRNA or complementary RNA (cRNA) analysis. The PCR conditions were 50 °C for 2 min, 95 °C for 2 min, and 45 cycles of 95 °C for 15 s, 55 °C for 30 s, and 72 °C for 30 s. Cycle threshold (Ct) values were normalized by the GAPDH RNA level.

### 2.12. Western Blotting

Cells for each of the conditions were collected and lysed using RIPA buffer. We used 10% sodium dodecyl sulfate polyacrylamide gel electrophoresis (SDS-PAGE) to separate the protein samples. After 1 h of blocking with 5% BSA, the protein bands were transferred to a PVDF membrane and cultured overnight with the following primary antibodies: PB2 (dilution 1:5000), NP (dilution 1:5000), kappaB p65 (dilution 1:1000), phospho-NF-κB p65 (dilution 1:1000), p38 MAPK (dilution 1:1000), phospho-p38 MAPK (dilution 1:1000), SOD3 (dilution 1:1000), Nrf2(dilution 1:1000), and anti-Flag (dilution 1:1000). GAPDH or β-actin was used as a protein control. The PVDF membrane was washed three times with washing buffer before being incubated for 1 h at room temperature with anti-rabbit or anti-mouse horseradish peroxidase (HRP)-conjugated secondary antibodies (dilution 1:1000). The protein bands were visualized using FluorChem E imaging system (ProteinSimple, Silicon Valley, CA, USA).

### 2.13. Immunofluorescence Staining

Intracellular localization of NP was assessed in PR8- infected A549 cells at 6 h p.i. by immunofluorescence staining with anti-NP mouse mAb (dilution 1:500). Additionally, we then incubated with FITC-labeled rabbit secondary antibody (dilution 1:250). Nuclei were stained using 49,6-diamidino-2-phenylindole (DAPI) (Sigma-Aldrich Chemie GmbH, Munich, Germany). The assay was observed with confocal laser scanning microscope (LSM 880 with Airyscan, Carl Zeiss Jena, Oberkochen, Germany) and a Ti-Eclipse inverted fluorescence microscope (Nikon, Tokyo, Japan).

### 2.14. Mini-Replicon Assay

293T cells grown in a 24-well plate were transfected with 50 ng NP and viral polymerase plasmids (pHW2K NP, pHW2K PA, pHW2K-PB1, pHW2K-PB2), pPolI Fluc (firefly luciferase reporter plasmid), and 10 ng hRluc TK (Renilla luciferase plasmid) with Lipofectamine 3000, as previously described [29]. The supernatant was replaced with fresh DMEM containing 10% FBS and continued culturing for 24 h for the subsequent experiments. The cells were lysed by cell lysates (Promega) for 20 min and the luciferase expression level was determined by dual-luciferase reporter assay system (Promega, Madison, WI, USA).

### 2.15. Apoptosis Detection

Apoptosis was assessed by a CaspaseAssay (Promega-Glo 3/7 Assay) following the manufacturer’s instructions. Briefly, the control cells and infected cells were washed with PBS, separated with 0.25% EDTA-free trypsin, and then rotated. The cells were centrifuged at 1500 rpm for 5 min and washed again with PBS. FITC Annexin V apoptosis detection kit and BD FACS Canto II flow cytometer (San Jose, CA, USA) were used to investigate cell apoptosis. The data were analyzed using FlowJo software (Treestar, San Carlos, CA, USA). At least 10,000 cells were analyzed. Mean fluorescence intensity is reported as percent increase over control cells.

### 2.16. SOD3 Expression In Vivo

Three-to-four-week-old male BALB/c mice were purchased from the laboratory animal center of southern medical university. Experimental mice were acclimatized in the experimental environment for one week before starting the experiment. IAV A/PR/8/34 (H1N1) murine lung-adapted strain used for vivo evaluation was donated by Prof. Yang Zifeng laboratory at Guangzhou Medical University. Our laboratory amplified SPF chicken embryos and stored them at -80℃. Three mice per group were employed in the following experiments. Mice were challenged with A/PR/8/34 (H1N1) by intranasal instillation. Virus group mice were delivered in 50 μL A/PR/8/34(5 LD_50_), while the blank control group was given equal amount of PBS solution. On 3rd day and 5th day p.i., the mice were sacrificed, and we retained mice lungs for subsequent experiments. The Western blotting procedure was as described above. RNA was extracted from lungs using total RNA Isolation Kit (Foregene, Chengdu, China) according to the manufacturer’s instructions. The primers used in this study are listed in Table S2.

### 2.17. Statistical Analysis

The results were expressed as mean ± SD of at least three independent experiments. The statistical analysis of all data was determined by two-tailed Student’s *t*-test with GraphPad Prism 8 software. * indicated *p* < 0.05, ** indicated *p* < 0.01, and *** indicated *p* < 0.001.

## 3. Results

### 3.1. ROS Production by IAV-Stimulated A549 Cells Was Time-Dependent

**Figure 1:**
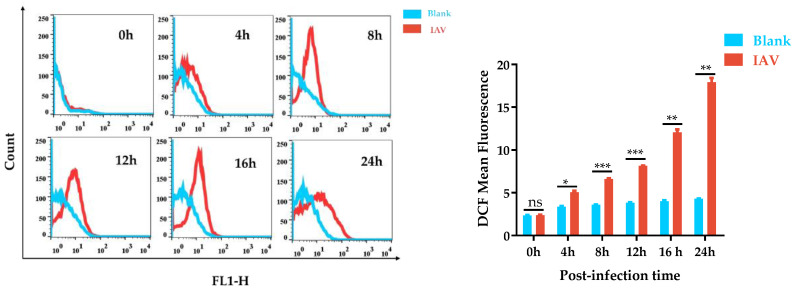
IAV-infection induced intracellular ROS production. Mean DCF fluorescence was determined by flow cytometry at various timepoints after IAV infection. * *p* < 0.05, ** *p* < 0.01, and *** *p* < 0.001; ns means *p* > 0.05.

Oxidative stress is caused by an imbalance between the production and accumulation of oxygen reaction products (ROS) in cells and tissues and the body’s ability to eliminate these reaction products. Therefore, the expression level of ROS can to some extent reflect the degree of oxidative stress. To determine whether IAV infection induced intracellular ROS production, the levels of intracellular ROS were measured with DCF staining at different timepoints following IAV infection (0, 4, 8, 12, 16, and 24 h). As shown in Figure 1, IAV infection caused a significant increase in the intracellular ROS generation appearing 4 h post infection (p.i.) compared with untreated cells, which extended to 24 h p.i. The H_2_O_2_ treatment group was used as a positive control, and a significant increase in intracellular ROS expression was also detected (Figure S1). The data indicated that IAV infection significantly and time dependently enhanced the production of ROS in infected A549 cells. The infected cells continuously created ROS to boost antioxidant defenses; however, excessive ROS may contribute to intensive oxidative stress.

### 3.2. IAV Infection Induces Oxidative Stress and Decreases Antioxidant Enzyme Activities

**Figure 2:**
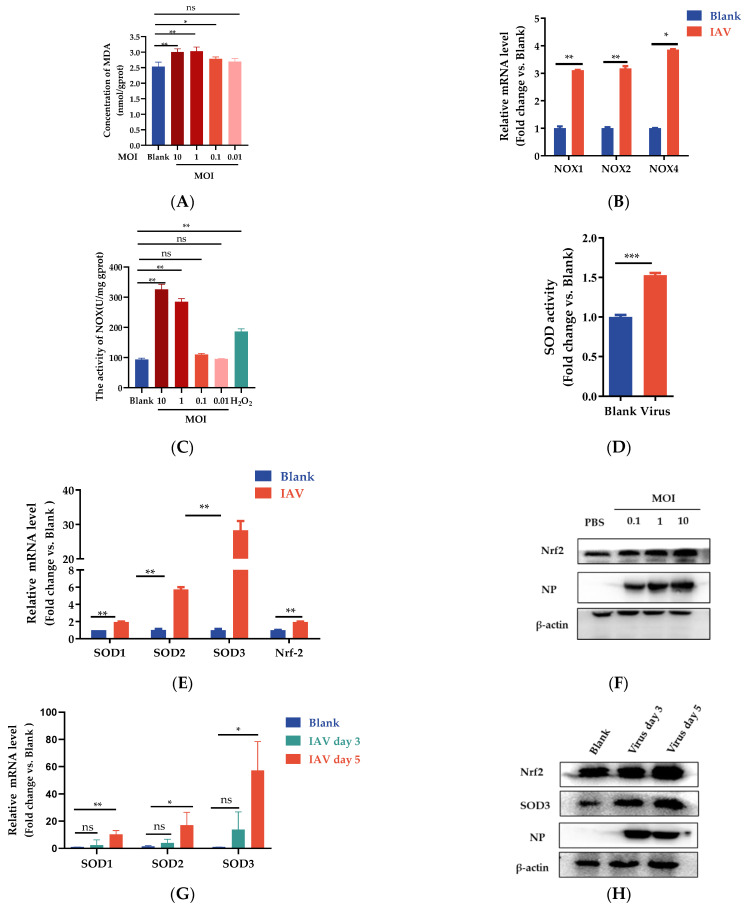

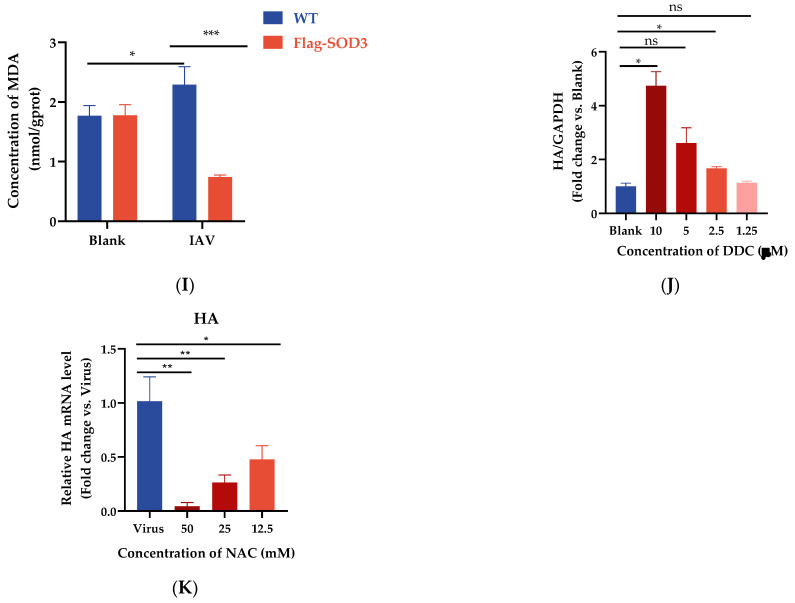
IAV infection modified the expression of oxidant and antioxidant enzymes. (**A**) The concentration of MDA in A549 cells was measured following 24 h of treatment of IAV at various MOIs; (**B**) the mRNA expression level of NADPH gene; (**C**) the activity of NADH oxidase activity; (**D**) total SOD activity in A549 cells was measured following 24 h of treatment of IAV at MOI of 1; (**E**) the mRNA expression level of SOD1, SOD2, SOD3, and Nrf-2 genes; (**F**) the Nrf2 protein level in A549 cells with treatment of IAV at MOI of 1; (**G**) the mRNA expression levels of SOD1, SOD2, and SOD3 in vivo; (**H**) the SOD3 and Nrf2 protein levels in vivo; (**I**) MDA levels in SOD3 overexpression group cells or WT group cells were measured following 24 h of IAV treatment at an MOI of 1; (**J**) HA mRNA production of the infected cells was detected by quantitative real-time PCR at 24 h post infection after treatment with different concentrations of DDC in virus-infected cells; (**K**) the HA mRNA level was detected after treatment with different concentrations of NAC in virus-infected cells. Data are shown as mean ± SEM. * *p* < 0.05, ** *p* < 0.01, and *** *p* < 0.001; ns means *p* > 0.05.

Since IAV infection induces an increase in ROS production and causes oxidative stress, we investigated whether IAV infection modifies the expression of oxidant and antioxidant enzymes. MDA as a biomarker is widely used for examining oxidative stress in the biomedical field [30]. We then measured the levels of MDA in A549 cells exposed to IAV at various MOIs. The result showed that A549 cells infected at a higher MOI (MOI = 10, 1, 0.1) showed a substantial increase in the MDA level compared with the control cells (*p* < 0.05); however, no significant difference was seen in the A549-infected cells at low MOI conditions (MOI = 0.01). These results suggested that IAV infection can induce oxidative stress in A549 cells, which is highly controlled by MOI (Figure 2A). NOX oxidases are a family of enzymes that generate ROS [31,32]. We also wanted to find out whether IAV infection induces a higher expression of NOX oxidases As shown in Figure 2B, the mRNA levels of NOX1, NOX2, and NOX4 oxidases were significantly increased in the PR8-infected cells compared with the control cells. Furthermore, we used an enzyme activity detection kit to detect the activity of NOX oxidase in A549 cells infected with different MOIs. The H_2_O_2_ treatment group was used as a positive control to induce cellular oxidative stress. The results demonstrated that A549 cells infected with IAV at a high MOI level showed a substantial increase in the NOX level compared with the control cells (Figure 2C).

The SOD family is known to play an important role in oxidative stress modulation. To measure SOD activities, total lysates were prepared from uninfected or infected A549 cells at 24 h p.i. As shown in Figure 2D, total SOD activity was significantly increased upon IAV infection compared with the uninfected cells (*p* < 0.001). Compared with uninfected cells, IAV infection was characterized by significantly lower SOD1 expression (*p* < 0.05) and higher SOD2 (*p* < 0.05) and SOD3 (*p* < 0.05) expression in the infected A549 cells. Moreover, SOD3 was markedly higher compared with SOD1 and SOD2. Furthermore, mRNA expression of nuclear factor E2-related factor 2 (Nrf2) increased upon viral infection, indicating that host cells can protect themselves from infection by promoting the expression of antioxidative enzymes (Figure 2E). Nrf2 changes in protein levels were measured by Western blot at 24 h post infection. The protein levels of Nrf2 also increased significantly after IAV infection. As a master regulator of antioxidative responses, the expression of Nrf2 has a positive correlation with the viral titer (Figure 2F). Similar results were confirmed in BALB/C mice infected with PR8 virus. Compared with the blank group, the mRNA levels of SOD1, SOD2, and SOD3 were significantly increased on the fifth day after PR8 infection (Figure 2G). The protein levels of SOD3 and Nrf-2 also increased significantly following the enhanced expression of IAV nucleoprotein (NP) (Figure 2H). These results also explained the increase in intracellular ROS in response to IAV infection. In comparison with the WT group cells, the SOD3 overexpression group cells showed dramatically reduced MDA levels in A549 cells (Figure 2I), indicating that SOD3 overexpression can counteract the oxidative stress caused by viral infection. Sodium diethyldithiocarbamate (DDC) is a superoxide dismutase (SOD) inhibitor. A concentration-dependent increase in HA mRNA expression was observed in infected cells treated with DDC at the indicated concentrations (Figure 2J). N-acetylcysteine (NAC) is one of the most classic antioxidants, which is used as a positive control to examine its effect on ROS accumulation [33]. Our results showed the effect of NAC is similar to the SOD3. Compared with the IAV group, NAC treatment could significantly reduce the replication of the virus (Figure 2K). These results implied that there is a potential relationship between SOD and IAV infection. Based on the above results, we hypothesize that the antioxidative enzyme SOD3 may be positively associated with cellular antiviral defenses.

### 3.3. SOD3 Disrupted IAV Replication in A549 Cells

**Figure 3:**
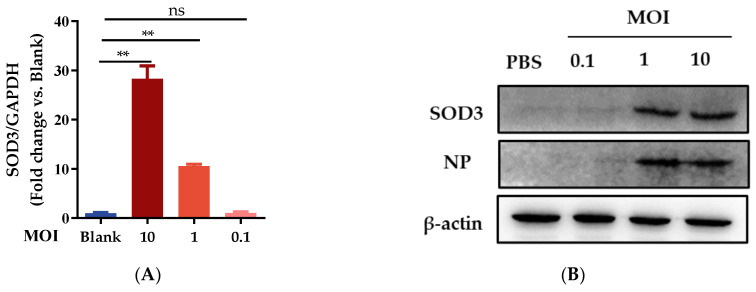

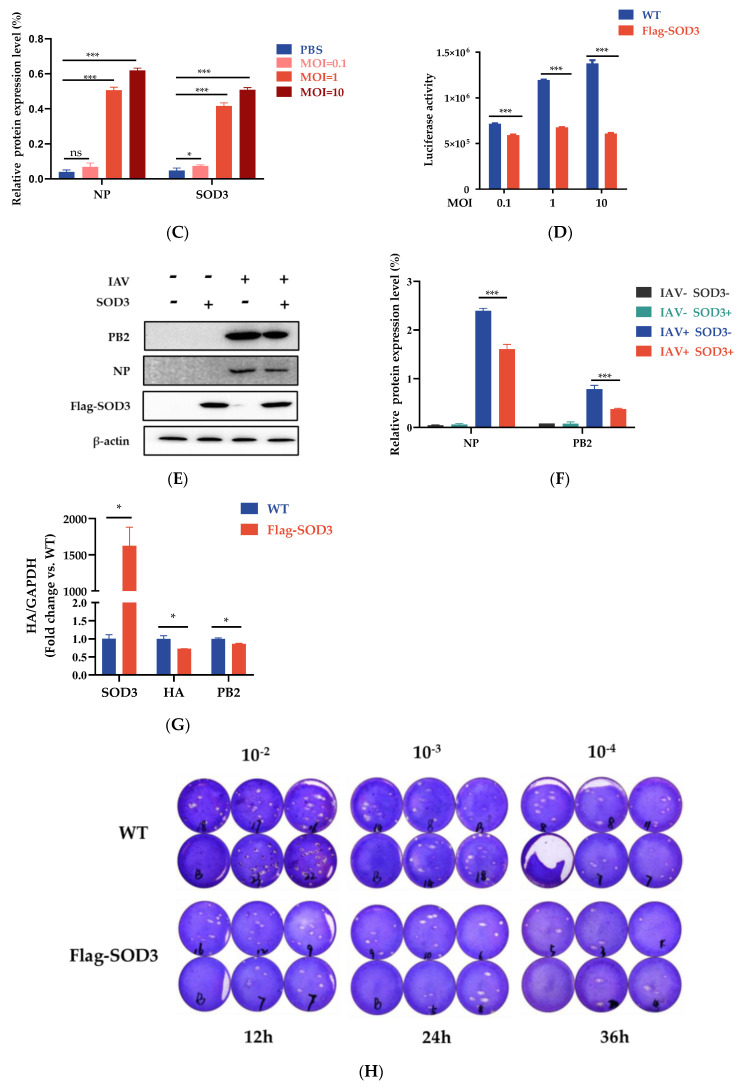

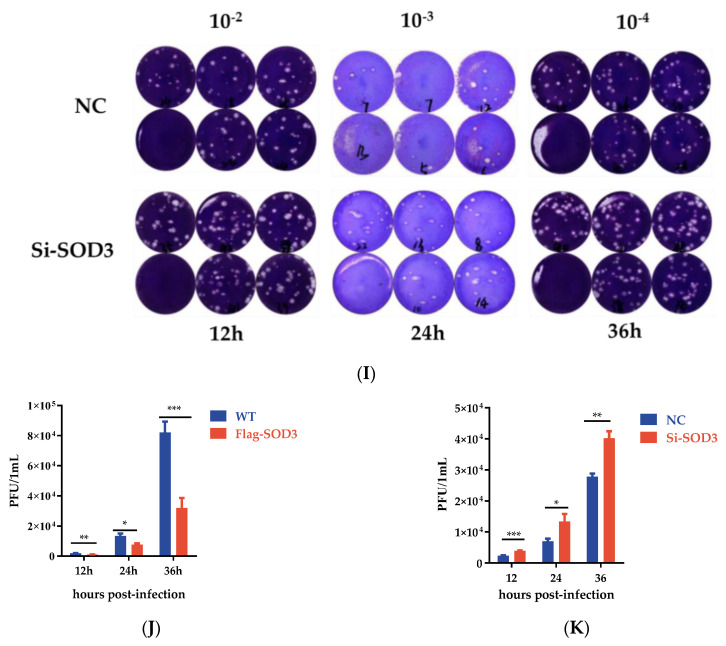
Influence of SOD3 on IAV replication in A549 cells; (**A**) relative mRNA expression of SOD3 under different MOI levels of virus infection; (**B**,**C**) protein abundance of SOD3 and NP in IAV-infected A549 cells; (**D**) pPoll-flu plasmid luciferase activity; (**E**,**F**) effect of SOD3 overexpression on influenza NP and PB2 proteins expression; (**G**) relative mRNA level of SOD3, HA, and PB2 genes; (**H**,**J**) effect of SOD3 overexpression on progeny virus production; (**I**,**K**) effect of SOD3 knockdown on progeny virus production (n = 3). All values are presented as means ± SEM. * *p* < 0.05, ** *p* < 0.01, and *** *p* < 0.001; ns means *p* > 0.05.

To determine if SOD3 is involved in IAV replication, we first evaluated SOD3 expression upon IAV challenge at different MOI levels. mRNA expression levels and protein expression were determined by qRT-PCR and Western blot assay, respectively. As presented in Figure 3A–C, we found that MOIs of 0.1, 1, or 10 actively induced the expression of SOD3 in PR8-infected A549 cells with the increase in NP protein or gene expression. These results suggested that SOD3 was involved in the modulation of influenza virus infection. To determine the impact of SOD3 on IAV replication, SOD3-expressing plasmid and pPoll-flu plasmid were co-transfected into A549 cells followed by PR8 infection at different MOIs. Overexpression of SOD3 significantly reduced the viral titer of PR8 (Figure 3D) and inhibited the expression of viral NP and PB2 proteins under IAV infection (Figure 3E,F). In addition, overexpression of SOD3 also inhibited the expression of HA and PB2 mRNA levels in HEK293 cells (Figure 3G). To examine the influence of SOD3 on virus progeny, A549 cells were infected at MOI 1, and infectious virus titers were determined by plaque assay at 12, 24 h, and 36 p.i. The plaque reduction experiment revealed that the overexpression of SOD3 significantly reduced infectious viral outputs at 24 h p.i., and they continued to decline at 36 h p.i. (Figure 3H,J). Conversely, when SOD3 was knocked down by short interfering RNA (siRNA) in A549 cells, virus proliferation was promoted over several growth cycles (Figure 3I,K), which suggests that SOD3 is an essential antiviral component in the suppression of IAV infections.

### 3.4. SOD3 Disrupted IAV Replication by Impairing the Synthesis of Viral RNA

**Figure 4:**
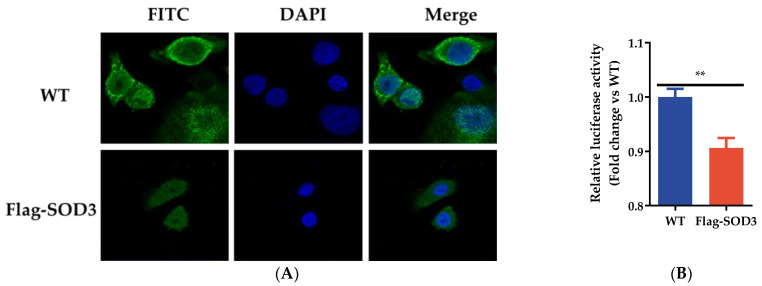
Effect of SOD3 on viral RNA generation. (**A**) Effect of SOD3 overexpression on the nuclear export of NP; (**B**) the effects of SOD3 on viral vRNP activity. ** *p* < 0.01.

Previous studies have demonstrated influenza ribonucleoprotein (RNP) is exported from the nucleus to the cytoplasm between 6 and 8 h after viral infection [34]. To explore how SOD3 impairs IAV replication, we examined the influence of SOD3 on the retention of RNP complexes. A549 cells were infected with PR8 (MOI = 0.1). Cells were analyzed for RNP exports at 6 h p.i. by labeling the NP of the IAV. Immunofluorescence staining revealed that SOD3 did not interfere with influenza RNP export from the nucleus to the cytoplasm (Figure 4A). To determine the effects of SOD3 on viral ribonucleoproteins (vRNP) activity, we conducted a mini-genome assay using four viral protein-expressing plasmids (pCAGGS) encoding PB1, PB2, PA, and NP, and the viral-like genome expression plasmid NP-luc/pPolI encoding firefly luciferase. As shown in Figure 4B, SOD3 significantly reduced viral polymerase activity.

vRNP is responsible for the transcription and replication of the influenza virus RNA genome. To further investigate whether SOD3 affects viral genome transcription, we assessed the level of IAV mRNA, vRNA, and cRNA by qRT-PCR analysis. In Flag-SOD3 group cells, the levels of vRNA and mRNA expression were significantly lower (at 9 p.i. and 24 h p.i., respectively) than those of the WT group cells (Table 1 and Table 2). Conversely, interfering SOD3 expression significantly increased vRNA expression at 9 h p.i. compared with the control group (Table 3). These results indicated that the overexpression of SOD3 may interfere with vRNA accumulation.

### 3.5. Effects of SOD3 on ROS Production in IAV-Induced A549 Cells

**Figure 5:**
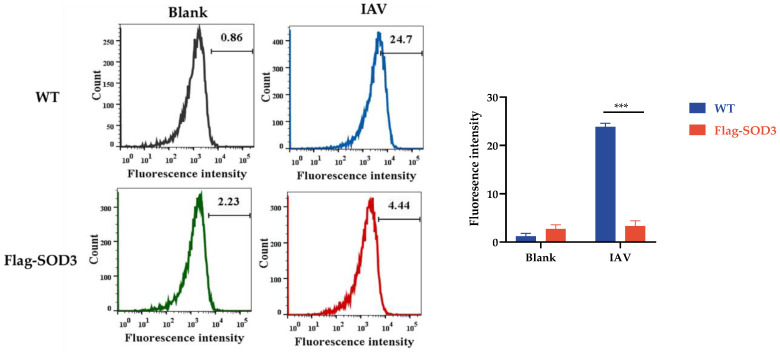
Overexpression of SOD3 reduced IAV-induced ROS production. ROS levels in A549 cells were analyzed by DCFH-DA via flow cytometry (n = 3). Data are expressed as the mean ± SEM. *** *p* < 0.001.

Given that SOD3 is a member of the antioxidant enzyme family, we investigated whether increasing SOD3 expression could reduce IAV-induced ROS production. The results of flow cytometry revealed that IAV increased intracellular ROS generation in A549 cells. Compared with the viral group, the level of ROS in the SOD3 overexpression group was significantly reduced (Figure 5). N-acetyl-L-cysteine (NAC) is a known antioxidant [35]. NAC (10 mg/mL) treatment could also significantly reduce the level of oxidative stress induced by H_2_O_2_ (Figure S2). Collectively, the mechanism by which SOD3 interferes with IAV replication included directly interacting with viral genome RNAs and inhibiting the IAV-induced formation of ROS.

### 3.6. SOD3 Overexpression Reduced Cellular Inflammation and Apoptosis Induced by IAV

**Figure 6:**
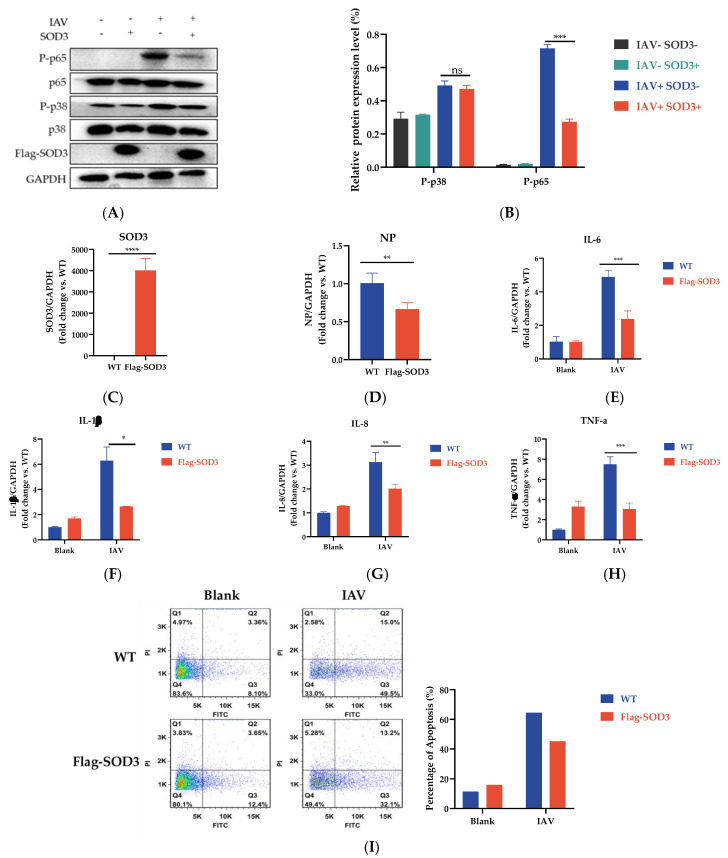
SOD3 overexpression inhibited cellular inflammation and apoptosis induced by IAV; (**A**,**B**) protein abundance of p65, pp65, p38, pp38, and SOD3 in A549 cells; (**C**) relative mRNA level of SOD3 gene; (**D**) relative mRNA level of NP gene; (**E**) relative mRNA level of IL-6 gene; (**F**) relative mRNA level of IL-1β gene; (**G**) relative mRNA level of IL-8 gene; (**H**) relative mRNA level of TNF-a gene; (**I**) cells apoptosis in A549 cells. Data are expressed as the mean ± SEM. * *p* < 0.1, ** *p* < 0.01, and *** *p* < 0.001; ns means no significance.

When ROS are produced excessively, specific oxidant-sensitive pathways, such as NF-κB and MAPK, are activated and have been reported to be related to influenza virus replication and pro-inflammatory response [36,37]. Further investigations were required to define whether SOD3 regulates viral replication through these signaling pathways. The results showed that the SOD3 overexpression reduced the phosphorylation levels of p65. SOD3 overexpression slightly reduced the expression of phosphorylated p38; however, the differences were not significant. (Figure 6A,B). Consistent with the above results, we found that the overexpression of SOD3 suppressed the expression of the IAV NP gene (Figure 6C,D), which further reduced the release of pro-inflammatory cytokines (IL-6, IL-8, TNF-α, and IL-1β) in response to IAV infection (Figure 6E–H). In conclusion, overexpression of SOD3 inhibited the expression of key phosphorylation proteins in the NF-κB signaling pathway and ultimately attenuated the production of the inflammatory factors induced by IAV infection.

ROS burst is often related to virus-induced apoptosis. We explored whether ROS elimination by SOD3 overexpression could restore IAV-induced apoptosis. WT cells or SOD3-overexpressed A549 cells were infected with PR8 at 1 MOI. After 24 h infection, the rate of apoptosis was evaluated by flow cytometry. The result indicated that SOD3 overexpression decreased virus-induced apoptosis (Figure 6I). NAC (10 mg/mL) was applied as the positive control to examine the effects of immune pathway signaling and apoptosis in IAV-infected cells. NAC could not only decrease the virus-induced phosphorylation of P38 and P65 (Figure S3A,B) but also reduce the transcription level of related inflammatory factors produced by virus stimulation, such as IL-6, IL-8, IL-1b, and TNF-a (Figure S3C–F). Furthermore, the addition of NAC could effectively inhibit the apoptosis caused by IAV infection (Figure S3G).

## 4. Discussion

When host cells suffer from a viral infection, ROS are spawned. A moderate increase in ROS can promote cell proliferation and differentiation [38], whereas excessive amounts of ROS can cause oxidative damage to cellular components and cell death. Some research has shown that influenza virus infection weakens antioxidant defense systems, as evidenced by massive ROS generation, consequently causing significant oxidative stress [18]. ROS overproduction has been shown to contribute to the development of influenza-virus-induced acute pulmonary damage [39]. In our experiment, a continuous increase in intracellular ROS production was observed after IAV infection compared with the control group, implying that cell defenses against IAV were strengthened. However, high levels of ROS can cause oxidative damage. To investigate whether oxidative stress induced by IAV infection is related to the MOI of the virus, we then measured levels of the oxidative stress biomarker MDA in A549 cells exposed to different MOI of IAVs. The results demonstrated that a high MOI of IAV exposure significantly increased MDA content compared with the uninfected cells. Following IAV infection, the expression of NOXs also increased and altered redox signaling. Our results were consistent with previous reports that IAV infection causes an oxidant stress response in A549 cells [40,41].

Although SOD3 was reported to minimize influenza-induced lung injury in mice by reducing oxidative stress [42], the role of SOD3 against pathogen invasion and the mechanisms governing SOD3′s response to influenza virus infection are not well understood. Thus, the detailed relationship between SOD3 and viral propagation must be determined. An increased understanding of SOD3 will greatly improve our knowledge of influenza virus pathogenesis and provide insight into the development of candidate antiviral therapeutics.

Infection with IAV may result in dramatic changes in the intracellular environment. Therefore, we analyzed the SODs lever, as well as some of the major cellular antioxidant enzymes (Nrf-2). We observed that the gene expression profiles of each of the three SODs differed when the target cells were infected with IAV. The result clearly demonstrated that IAV infection enhanced the level of SODs. SOD3 was the most highly expressed for the duration of IAV infection compared with the expression of SOD1 and SOD2. Except for the result related to environmental and pathogenic stress, SOD3 augmentation may have a specific role in combating IAV infection. This underlying mechanism should be further investigated.

SOD3 is known to modulate environmental oxidative stress by catalyzing the dismutation of O^2−^ to H_2_O_2_ and O_2_. Little is known about the regulatory role of SOD3 in IAV replication. When SOD3 was highly upregulated during IAV infection, the virus titers were remarkably decreased. Furthermore, SOD3 overexpression significantly decreased viral titers. This finding suggested that an increase in SOD3 may be to blame for the decrease in viral yield. SOD3 deficiency, on the other hand, increased the yield of progeny viruses and promoted the formation of virus-induced plaque. These findings support our hypothesis that SOD3 is implicated in virus replication as an important antiviral factor in the pathogenesis of IAV infection in addition to attenuating oxidative stress. Our results also prompted further exploration of the relationship between SOD3 and virus yield.

During influenza virus replication, the influenza RNA-dependent RNA polymerase (RdRP) is bound to newly synthesized vRNAs and is further wrapped with NP to form vRNP in the host cell nucleus, then it is subsequently exported into the cytosol to be assembled with the other structural proteins for packaging into progeny virions [43]. vRNP is crucial for virus replication. Previous studies have found that the efficient retention of vRNP complexes in the nuclei of infected cells inhibit IAV replication [44]. Since N-acetyl-L-cysteine (NAC) as a known antioxidant has been shown to influence seasonal IAV replication via blocking the nuclear export of vRNP [45], we speculated that SOD3 might interfere with the nuclear export of vRNP to inhibit virus replication. Noticeably, SOD3 had no marked effect on the export of vRNP from the nucleus to cytoplasm. The data highlight that forced SOD3 expression disrupts IAV replication through unknown mechanisms, which seemed closely related to direct antiviral activity.

We further evaluated the influence of SOD3 on vRNP activity by using a mini-replicon assay. The results effectively demonstrated that the polymerase activity was greatly decreased in SOD3-overexpressed cells compared with the control cells. Influenza virus RNA synthesis consists of three steps: (I) the transcription of vRNAs into mRNA, (II) replication of vRNA into cRNA, and (III) replication of cRNA into vRNA [46]. Both vRNA and cRNA are thought to be created by replicas, although their synthesis is distinct. According to the relevant research, vRNA synthesis begins internally, and the developing vRNA is subsequently rearranged to the end of the cRNA promoter, whereas cRNA synthesis is initiated at the end of the vRNA promoter [47]. Although the three different types of RNA have been extensively studied [48], the regulatory mechanism governing the production of the three distinct viral RNAs in virus-infected cells remains unclear. Our study showed that SOD3 preferentially regulates the synthesis of vRNA rather than the synthesis of complementary positive-strand cRNA or mRNA, which contributes to the effective replication of the influenza virus. SOD3’s differential regulation of influenza virus RNA synthesis may play a key role in managing the amount of the three viral RNA types in infected cells to control virus RNA and protein synthesis, and in this way, minimize virus production.

The NF-kB and MAPK pathways are two important pathways that are involved in IAV replication and pro-inflammation response. Both are also specifically oxidant-sensitive pathways [36]. Therefore, we investigated the effects of SOD3 overexpression on cellular signaling pathways associated with IAV infection. Our results showed that SOD3 overexpression reduced the PR8-induced phosphorylation of p65 and drastically decreased the transcription of several pro-inflammatory cytokines, including IL-6, IL-8, TNF-α, and IL-1β.

Apoptosis usually involves mitochondrial damage, ROS production, and oxidative stress. Decades of research have shown that ROS are a very important regulatory upstream factor in cell apoptosis. Mitochondria-dependent and mitochondria-independent apoptosis pathways are regulated by ROS [49]. The preliminary findings of our study showed that overexpression of SOD3 could inhibit the early apoptosis of cells. Combined with the above experimental results, our preliminary hypothesis was that SOD3 overexpression inhibits the infection of IAVs and thereby reduces the level of ROS in cells, which further inhibits cell apoptosis caused by ROS. However, the specific mechanism of SOD3 to inhibit ROS production and its transformation into anti-apoptotic signals remains unclear. Further research is needed to explore this mechanism.

Here, we emphasize the antiviral and antioxidative activities of SOD3 as a major antioxidant enzyme. However, our study suffers from some limitations. Investigating the underlying mechanism using SOD3 transgenic mice will shed light on the in vivo role of SOD3 in inhibiting influenza proliferation. With further studies, SOD3 will prove to be an important host factor that is closely associated with the pathogenesis of the influenza virus. Our study revealed that SOD3 has a biphasic effect on IAV infection. SOD3 not only plays a key role in cellular defense against IAV infection by eliminating ROS but also directly impairs IAV replication by inhibiting viral vRNA. Our results further revealed the role of SOD3 in antiviral innate immune responses to influenza virus infection and the role of SOD3 as a novel target for influenza prevention and treatment.

## 5. Conclusions

In conclusion, our results revealed the potential role of SOD3 in resistance to IAV infection. We confirmed that SOD3 is remarkably enhanced after IAV challenge. SOD3 is involved in IAV replication by regulating vRNA synthesis. Moreover, SOD3 alleviates the pro-inflammatory response, inhibits apoptosis, and protects cells against oxidative stress damage. This study has taken steps towards revealing the novel mechanisms by which SOD3 alleviates oxidative stress in IAV infection and may contribute to its future application in influenza virus-related diseases.

## Figures and Tables

**Table 1:** SOD3 overexpression specifically reduced vRNA levels at 9 h p.i.

RNA Species	RNA Segment	Levels after Treatment with:		CT Difference	*p* Value
		WT	Flag-SOD3		
vRNA	NA	18.96 ± 0.47	19.67 ± 0.11	0.71	0.0249
	NP	16.52 ± 0.40	17.22 ± 0.34	0.70	0.0379
	PB1	19.73 ± 0.16	20.29 ± 0.02	0.56	0.0004
	PA	16.42 ± 0.33	16.93 ± 0.05	0.51	0.0209
	PB2	18.02 ± 0.09	19.03 ± 0.07	1.01	<0.0001
	NS	17.78 ± 0.13	18.67 ± 0.12	0.89	<0.0001
	M2	18.33 ± 0.14	19.16 ± 0.11	0.83	<0.0001
	HA	21.85 ± 0.20	23.12 ± 0.40	1.27	0.0013
cRNA	NA	22.27 ± 0.45	22.20 ± 0.12	−0.07	0.7726
	NP	19.79 ± 0.22	19.78 ± 0.24	−0.01	0.9451
	PB1	20.81 ± 0.11	20.89 ± 0.09	0.08	0.2922
	PA	20.13 ± 0.40	19.76 ± 0.06	−0.37	0.1171
	PB2	20.79 ± 0.06	20.90 ± 0.08	0.11	0.0654
	NS	21.80 ± 0.13	21.96 ± 0.10	0.16	0.1014
	M2	20.84 ± 0.07	20.96 ± 0.06	0.12	0.0474
	HA	26.12 ± 0.29	26.44 ± 0.32	0.32	0.1777
mRNA	NA	17.42 ± 0.28	17.63 ± 0.14	0.21	0.234
	NP	13.51 ± 0.41	13.52 ± 0.19	0.01	0.9496
	PB1	16.83 ± 0.08	16.92 ± 0.13	0.09	0.2895
	PA	16.64 ± 0.07	16.71 ± 0.04	0.07	0.0952
	PB2	16.88 ± 0.08	17.21 ± 0.13	0.33	0.0047
	NS	14.82 ± 0.11	14.90 ± 0.09	0.08	0.31
	M2	15.45 ± 0.09	15.66 ± 0.10	0.21	0.0183
	HA	19.36 ± 0.33	19.49 ± 0.14	0.13	0.4828

**Table 2:** SOD3 overexpression specifically reduced vRNA levels at 24 h p.i.

RNA Species	RNA Segment	Levels after Treatment with:		CT Difference	*p* Value
		WT	Flag-SOD3		
vRNA	NA	20.02 ± 0.18	21.53 ± 0.14	1.51	0.0003
	NP	17.8 ± 0.01	19.12 ± 0.03	1.32	<0.0001
	PB1	20.91 ± 0.05	21.88 ± 0.03	0.97	<0.0001
	PA	18.78 ± 0.09	20.01 ± 0.02	1.23	<0.0001
	PB2	19.49 ± 0.1	20.91 ± 0.01	1.42	<0.0001
	NS	18.84 ± 0.17	20.12 ± 0.03	1.28	0.0002
	M2	19.62 ± 0.04	21.04 ± 0.05	1.42	<0.0001
	HA	23.79 ± 0.19	25.27 ± 0.19	1.48	0.0007
cRNA	NA	23.28 ± 0.25	24.22 ± 0.14	0.94	0.0047
	NP	20.09 ± 0.27	19.77 ± 0.17	−0.32	0.1563
	PB1	20.79 ± 0.13	21.41 ± 0.06	0.62	0.0016
	PA	20.15 ± 0.59	20.51 ± 0.12	0.36	0.3695
	PB2	20.51 ± 0.01	21.01 ± 0.07	0.5	0.0002
	NS	21.7 ± 0.06	22.21 ± 0.09	0.51	0.0012
	M2	20.59 ± 0.15	21.51 ± 0.04	0.92	0.0006
	HA	26.72 ± 0.39	27.54 ± 0.19	0.82	0.0312
mRNA	NA	20.34 ± 0.31	20.54 ± 0.25	0.20	0.4510
	NP	15.31 ± 0.25	15.58 ± 0.18	0.27	0.1908
	PB1	18.59 ± 0.13	18.85 ± 0.12	0.26	0.0684
	PA	18.69 ± 0.78	18.76 ± 0.08	0.07	0.8906
	PB2	18.38 ± 0.21	18.82 ± 0.11	0.44	0.0317
	NS	15.82 ± 0.11	16.32 ± 0.08	0.50	0.0029
	M2	16.56 ± 0.12	17.00 ± 0.08	0.44	0.0059
	HA	22.91 ± 0.63	23.32 ± 0.70	0.41	0.4953

**Table 3:** Knocking down SOD3 increased vRNA expression.

RNA Species	RNA Segment	Levels after Treatment with:		CT Difference	*p* Value
		NC	si-SOD3		
vRNA	HA	17.98 ± 0.2	16.08 ± 0.20	−1.90	<0.0001
	NA	19.03 ± 0.39	16.91 ± 0.10	−2.12	<0.0001
	NP	14.28 ± 0.15	12.72 ± 0.10	−1.56	<0.0001
	PB2	15.51 ± 0.26	14.08 ± 0.17	−1.43	<0.0001
	NS	14.42 ± 0.18	13.07 ± 0.04	−1.35	<0.0001
cRNA	HA	25.43 ± 0.1	26.00 ± 0.25	0.57	0.0078
	NA	24.7 ± 0.08	24.06 ± 0.30	−0.64	0.00600
	NP	20.85 ± 0.16	20.80 ± 0.17	−0.05	0.6697
	PB2	20.39 ± 0.14	20.95 ± 0.15	0.56	0.0017
	NS	21.08 ± 0.1	20.73 ± 0.19	−0.35	0.0168
mRNA	HA	22.37 ± 0.47	21.76 ± 0.07	−0.61	0.0424
	NA	20.71 ± 0.36	19.88 ± 0.09	−0.83	0.0044
	NP	16.84 ± 0.1	16.40 ± 0.19	−0.44	0.0063
	PB2	16.69 ± 0.11	16.45 ± 0.20	−0.24	0.0773
	NS	17.06 ± 0.27	16.73 ± 0.29	−0.33	0.1552

## Data Availability

All data are presented in the article and Supplementary Materials.

## Supplementary Materials

The following supporting information can be downloaded at: https://www.mdpi.com/article/10.3390/antiox12020354/s1; Figure S1: H2O2 treatment induced intracellular ROS production. Mean DCF fluorescence was determined by flow cytometry at various time points after H2O2 treatment; Figure S2: NAC treatment reduced H2O2-induced ROS production. ROS levels in A549 cells were analyzed by DCFH-DA via flow cytometry (n=3). Data are expressed as the mean ± SEM. *p* < 0.1, ** *p* < 0.001; Figure S3: NAC treatment inhibited cellular inflammation and apoptosis induced by IAV. (A, B) The expression of p65, Pp65, p38 and Pp38 in A549 cells. mRNA level of IL-6(C)，IL-1β gene(D)，TNF-α (E)，IL-8 (F) in virus-infected cells. (G) The effect of NAC in IAV induced apop-tosis. Data are expressed as the mean ± SEM. *p* < 0.1, * *p* < 0.01, *** *p* < 0.001, ns means no sig-nificance; Table S1: Primer Sequences for Quantitative Real-time PCR; Table S2: Primer Sequences for Quantitative Real-time PCR.
