# Supplementary material for: Potential Role of Superoxide Dismutase 3 (SOD3) in Resistance to Influenza A Virus Infection
Source: Antioxidants (Basel). 2023 Feb 2;12(2):354. doi: 10.3390/antiox12020354 (PMC9952479; doi:10.3390/antiox12020354)
Supplement: Supplementary file 1 [file antioxidants-12-00354-s001.zip › Revised supplementary Figures.pdf]

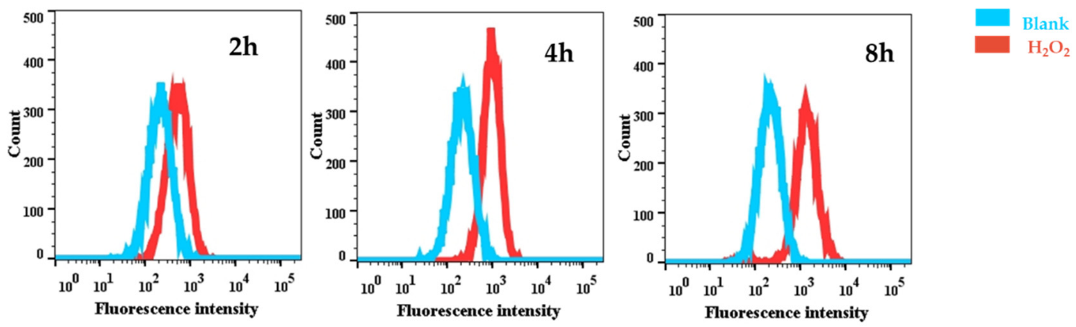

Figure S1. H_2O_2 treatment induced intracellular ROS production. Mean DCF fluorescence was determined by flow cytometry at various time points after H_2O_2 treatment.

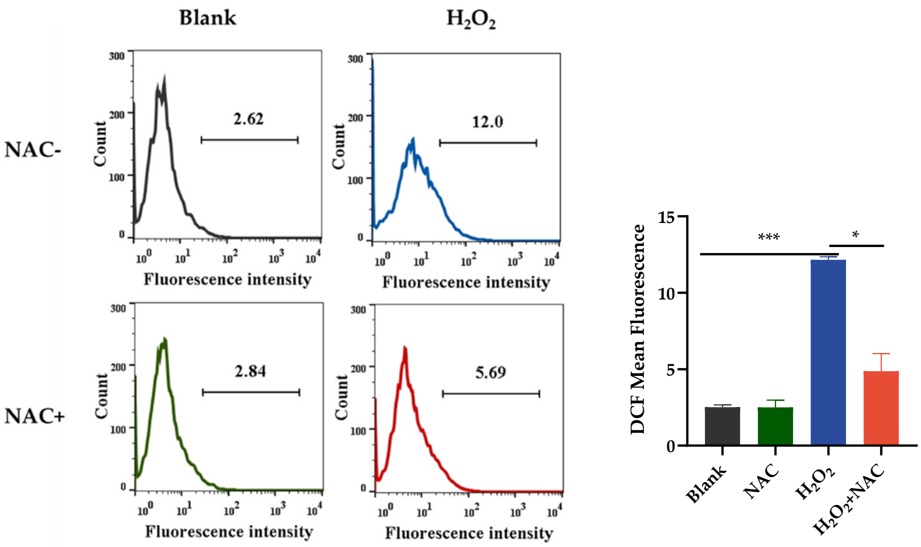

Figure S2. NAC treatment reduced H2O2-induced ROS production. ROS levels in A549 cells were analyzed by DCFH-DA via flow cytometry (n=3). Data are expressed as the mean ± SEM. *p<0.1, *** $p < 0.001$.

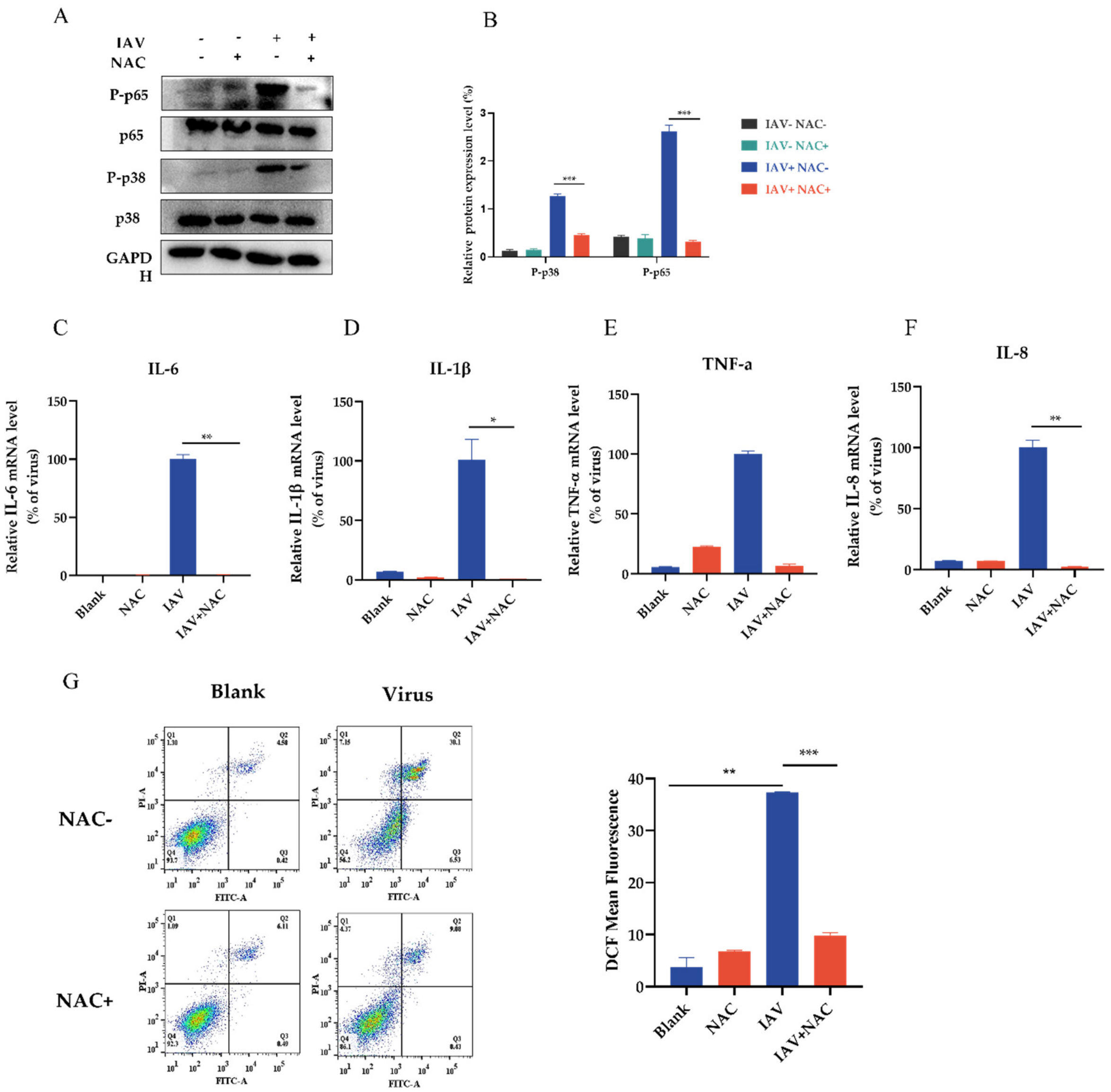

Figure S3. NAC treatment inhibited cellular inflammation and apoptosis induced by IAV. (A, B) The expression of p65, Pp65, p38 and Pp38 in A549 cells. mRNA level of IL-6(C), IL-1β gene(D), TNF-a (E), IL-8 (F) in virus-infected cells. (G) The effect of NAC in IAV induced apoptosis. Data are expressed as the mean ± SEM. *$p < 0.1$, ** $p < 0.01$, *** $p < 0.001$, ns means no significance.
